# Diagnosis of Lumbar Spondylolisthesis Using Optimized Pretrained CNN Models

**DOI:** 10.1155/2022/7459260

**Published:** 2022-04-13

**Authors:** Deepika Saravagi, Shweta Agrawal, Manisha Saravagi, Jyotir Moy Chatterjee, Mohit Agarwal

**Affiliations:** ^1^Department of Computer Application, SAGE University, Indore, MP 452012, India; ^2^IAC SAGE University Indore, MP 452012, India; ^3^Physiotherapy Department, Railway Hospital, Kota, Rajasthan 324002, India; ^4^Lord Buddha Education Foundation, Kathmandu, Nepal; ^5^Bennett University, Greater Noida, India

## Abstract

Spondylolisthesis refers to the slippage of one vertebral body over the adjacent one. It is a chronic condition that requires early detection to prevent unpleasant surgery. The paper presents an optimized deep learning model for detecting spondylolisthesis in X-ray radiographs. The dataset contains a total of 299 X-ray radiographs from which 156 images are showing the spine with spondylolisthesis and 143 images are of the normal spine. Image augmentation technique is used to increase the data samples. In this study, VGG16 and InceptionV3 models were used for the image classification task. The developed model is optimized by utilizing the TFLite model optimization technique. The experimental result shows that the VGG16 model has achieved a 98% accuracy rate, which is higher than InceptionV3's 96% accuracy rate. The size of the implemented model is reduced up to four times so it can be used on small devices. The compressed VGG16 and InceptionV3 models have achieved 100% and 96% accuracy rate, respectively. Our finding shows that the implemented models were outperformed in the diagnosis of lumbar spondylolisthesis as compared to the model suggested by Varcin et al. (which had a maximum of 93% accuracy rate). Also, the developed quantized model has achieved higher accuracy rate than Zebin and Rezvy's (VGG16 + TFLite) model with 90% accuracy. Furthermore, by evaluating the model's performance on other publicly available datasets, we have generalised our approach on the public platform.

## 1. Introduction

Spondylolisthesis, the most prevalent immature spine condition, is characterised by the anterior displacement of lumbar vertebrae relative to adjacent vertebrae. Spondylolisthesis affects around 4% to 6% of the population [1–3]. Early detection of spondylolisthesis using radiographs may prevent from surgery. Availability of large amounts of multimodal data in the healthcare domain prompted researchers to create and deploy Artificial Intelligence (AI) algorithms in this sector [4]. The importance of AI methods in the healthcare sector has increased dramatically in recent decades [5, 6].

Approaches for categorizing and detecting vertebral column diseases typically include image processing techniques. Image classification has long been a research hotspot, and Deep Learning (DL) methods provide a wide range of capabilities and flexibility that can be used in image classification [7]. Convolutional Neural Network (CNN) is the most popular type of Deep Neural Network (DNN) that uses multilayer pixel-based Artificial Neural Network (ANN) methods [8]. CNN contains one input layer, many hidden layers, and single output layer. It is widely used to classify images and outperform feature-based approaches in image classification, as well as giving promising results in medical imaging [9, 10].

To build a strong CNN model, a lot of labelled training data as well as excellent picture quality is required [11, 12]. ImageNet is a growing image database with 14 million pictures and 21841 synsets catalogued [13]. The ImageNet dataset has been used to construct a number of state-of-the-art CNN networks, including, VGG16 [11–14] and InceptionV3 [15].

In terms of the development of extremely popular pre-trained models for image classification, 2014 was a turning point. Two of the best models for image classification using Keras are VGG16 and InceptionV3 [16]. In that year's ILSVRC, VGG16 came in second place, while Google took first place with its model GoogLeNet (known as Inception now) [17]. Due to the popularity of these models, we have selected them for disease classification task in this study.

A private dataset comprising 299 spine X-ray images is used in this research. As there were less data to work with, data augmentation [18, 19] and transfer learning [20–22] approaches were used to increase sample size. These are significant methods to overcome the need for a large dataset in applications where data is limited, such as medical imaging.

To diagnose lumbar spondylolisthesis, two distinct CNN architectures, VGG16 and InceptionV3, were utilized in this study. TFLite is used to create a quantized model that requires less storage and offer a quick and accurate diagnosis of lumbar spondylolisthesis.

The major goals of the paper are as follows:

1. Implement a flexible, quick, and quantized pre-trained model to use on small devices and also compare the accuracy of the implemented algorithm with previous studies
2. Generalize the model on a public platform

This paper is organized as follows: (1) introduction about the need for image classification, (2) literature review, (3) overviews of selected pre-trained CNN algorithms for the diagnosis of spondylolisthesis, (4) materials and methods, (5) experimental setups, (6) result analysis and discussion, and (7) conclusion.

## 2. Literature Review

Many researchers have proposed solutions for healthcare domain applications by utilizing DL models.  Table 1 summarises all of the literature research discussed in this section.

Varcin et al. [23] used two well-known artificial neural networks, AlexNet and GoogLeNet, to solve the challenge of spondylolisthesis diagnosis. The model optimization technique is not used by the authors.

Cococi et al. [24] presented TensorFlow Lite for constructing intelligent medical devices by implementing MobileNetV3, ShuffleNetV2, and SlimNet models with Android to achieve a reasonable balance between accuracy and portability.

Cococi et al. [25] built an efficient recognition convolutional deep learning architecture integrated using Android and Raspberry Pi to run on portable, energy-efficient, resource-constrained platforms in the creation of intelligent medical equipment.

Basantwani et al. [26] have developed an Android application that employs a machine learning model to estimate COVID-19 in chest X-ray or CT scan. The final model was converted into a TFLite model which could be used in making the Android model.

Verma et al. [27] have created an innovative Android application that uses a very efficient and accurate DL algorithm to identify COVID-19 infection from chest CT images. The model generates a TensorFlow lite flat buffer file (.tflite) which is used to decrease the model's size, and the model is optimized for speed and latency on edge devices.

Bushra et al. [28] developed a CNN model and then converted it to TensorFlow Lite (TFLite) model to deploy on Android mobile.

Zebin and Rezvy [29] used multiple pre-trained convolutional backbones as the feature extractor to discriminate COVID-19 and Pneumonia-related inflammation in the lungs from normal inflammation.

Sharma et al. [30] have developed a model with the goal of detecting the existence of three pathologies, namely, Diabetic Macular Edema (DME), Choroidal Neovascularization (CNV), and Drusen and classified them using OCT (Optical Coherence Tomography).

We explored literature review based on models used in medical disease diagnosis using X-ray images because there is only one study in our field. After reviewing the literature, we have got that many researchers have utilized TFLite for model optimization technique for the diagnosis of different diseases based on an X-ray image dataset and achieved good accuracy (ranges between 90 and 99.38%).

## 3. Model Architecture

### 3.1. Pre-Trained VGG16 Model

VGG16 is a six-stage pre-trained model. Two convolution layers along with a max-pooling layer of stride 2 are used in the starting two stages. Three convolution layers with a max-pooling layer of stride 2 are used in the next three phases. Three fully connected layers make up the final stage. The convolution layers have a size of 3 × 3 filters with a stride of 1. Except for stage 5, each level doubles the number of filters starting at 64 [31–36].

**Figure 1:**
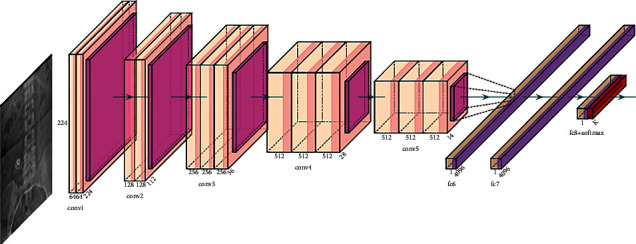
VGG16 architecture for spondylolisthesis diagnosis.

Figure 1 shows the architecture of VGG16 model which accepts spine X-ray image of dimension of 224 × 224 × 3 and after feature extraction the model is fine-tuned for binary classification of spondylolisthesis dataset.

### 3.2. Pre-trained InceptionV3 Model

The InceptionV3 network is made up of various modules that enable more efficient computation and deeper networks by using stacked 1 × 1 convolutions to reduce dimensionality. Some operations, such as 1 × 1, 3 × 3, and 5 × 5 convolutions and max pooling, are done in parallel and chained. “Inception layer” is the name given to this concatenation [15], [22], [37], [38].

**Figure 2:**
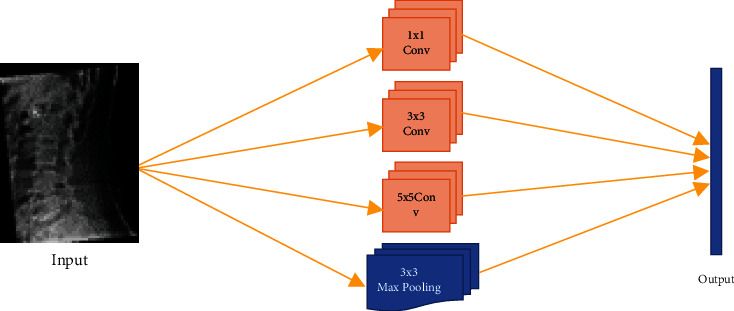
Inception layer architecture for spondylolisthesis diagnosis.

InceptionV3 model accepts spine X-ray image as input of dimension of 299 × 299 × 3 and after feature extraction and fine-tuning it gives the binary classification of spine X-ray image as output using SoftMax function.  Figure 2 explains the architecture of Inception layer used for spondylolisthesis dataset.

Some characteristics of cutting-edge pre-trained CNN networks, VGG16 and IncepsionV3, are shown in Table 2 [39, 40]. The selected models were preloaded with ImageNet weights and then fine-tuned for binary classification task. Categorical Cross-Entropy Loss (CE) is used to train both models.

## 4. Materials and Methods

**Figure 3:**
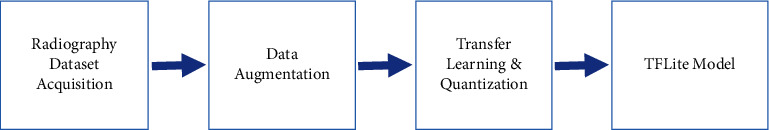
Block diagram of proposed work.

By optimizing and compressing the size of a pre-trained transfer learning model with TFLite, we were able to develop a quantized model which can be used on small devices.  Figure 3 illustrates the block diagram for the planned process.

In the first stage, radiographic images with the spine X-ray are taken and kept in the acquire-data stage. Data augmentation is employed to enhance the number of data samples in the second stage, and pre-trained models are used to extract decision features.

After training a partial dataset, proposed models are assessed for efficiency in the next stage. Then, using TFLite, the tested model is reduced up to four times to provide a lightweight, rapid, and accurate model for lumbar spondylolisthesis diagnosis.

### 4.1. Radiographic Image Acquisition

**Figure 4:**
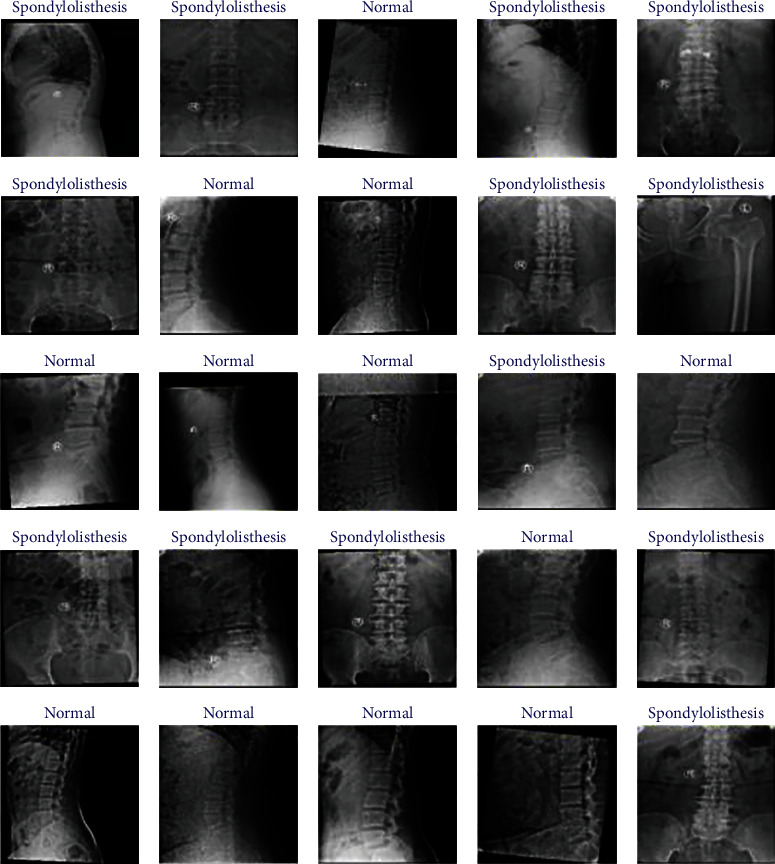
Glimpse of X-ray images from our private dataset.

A real-time dataset was collected from our own private collection of X-rays for this investigation. Physiotherapy and rehabilitation professionals categorized the collected radiographic images of the vertebrae as healthy or spondylolisthesis images. Some of the radiographs were removed because they were not technically sound. Some of the images from the dataset along with their classifications are shown in Figure 4.

The dataset contains a total of 299 spine X-ray images in various diameters. It includes 156 images of people with spondylolisthesis and 143 images of healthy people (without spondylolisthesis).

The radiographs were resized with 224 × 224 × 3 dimensions, in order to create images of vertebral columns mainly focusing on L4-L5 and L5-S1 vertebra [41].  Table 3 lists the features of the final dataset.

For effective prediction, all DL models require large sets of data. We utilized data augmentation to generate an adequate number of images from our dataset for proper diagnosis of disease.

### 4.2. Data Augmentation

In order to obtain adequate datasets, the data augmentation technique is used to generate more data based on image processing technologies. The original data from which the additional training data were generated are labelled in this augmentation approach[18, 19].

While improving overall performance, visual data augmentation prevents CNN from learning irrelevant patterns, overfitting, and retaining the specific properties of the training images. Cropping, translating, and reflecting the image are only a few of the data augmentation procedures. In this study, 701 additional images were created as a result of the data augmentation.

### 4.3. Transfer Learning

CNN model suffers from issues related to lack of dataset diversity and quantity. The goal of transfer learning is to impart knowledge in a domain by using a large amount of training data [12], [18].

### 4.4. Quantization Using TFLite

A native TensorFlow Lite quantization can be used to optimize a model [42]. It is used to transform the whole model into a flat buffer. A computer uses 32-bit floating-point representation of a real number for most purposes; quantization is a novel concept that transforms these 32-bit floating-point values to 8-bit integers with minor or no accuracy loss. This results in a huge reduction in the model's size [43].

**Figure 5:**
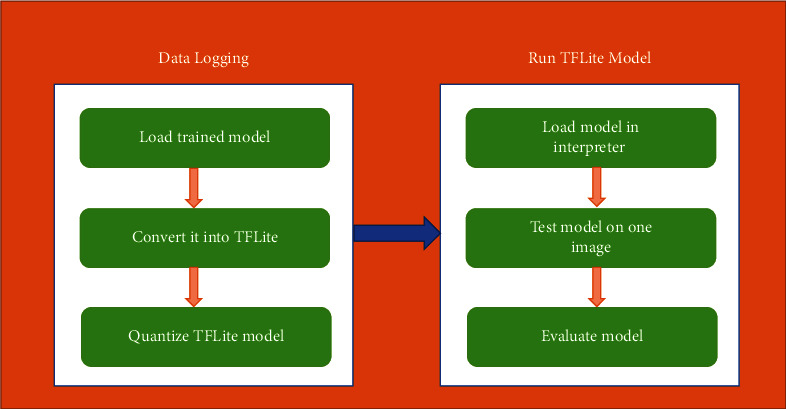
TFLite model compression process.

Figure 5 shows the entire process of model compression. The initial step is to log the data before compression. The trained model is converted into a TensorFlow Lite model using TFLite Converter. Then we set optimization flag = optimize to reduce the size of the resultant file.

In the second phase, we measure the performance of quantized (TFLite) model. The model is loaded into the interpreter to test it on a single image. Finally, the model is evaluated for whole dataset and the accuracies of base and quantized models were compared to check the difference.

## 5. Experiments

### 5.1. Experimental Design

This experiment was built on Python3 in a Windows environment using the Google Colab platform. The current version of TensorFlow, a DL framework, is 2.5.0. Accuracy/loss curves will be displayed using the pyplot module from the Matplotlib package, which offers a MATLAB-like interface to the underlying object-oriented charting library. To get the desired plot, it generates figures and axes implicitly and automatically.

#### 5.1.1. Data Splitting

Dataset is split into standard 70 : 30 ratio with 224 × 224 × 3 dimensions and separated into three groups, training set (700), test set (50), and validation set (250), using the train_test_split() method, with test step 50 and test batch 1.  Table 4 illustrates the statistics of split dataset.

#### 5.1.2. Model Training

The first step is to build a model that was created from a large number of datasets. The training set is used to train the model and test set is used to test the model for the image classification task. Test data is applied to determine the performance of the specified algorithms using the above mentioned training parameters.

### 5.2. Experimental Results

In this study, two pre-trained transfer learning models from classical and modern architectures were utilized (as explained in the Model Architecture section). The performance of each model is evaluated in terms of accuracy/loss graphs and confusion matrix.

#### 5.2.1. Training Accuracy/Loss

**Figure 6:**
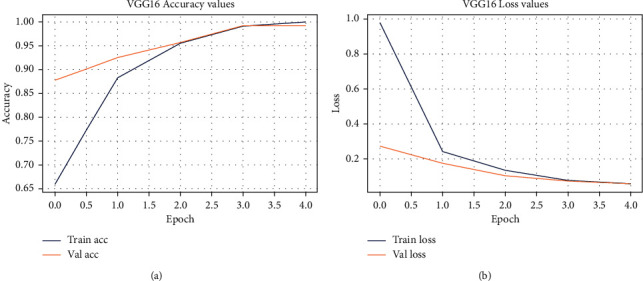
Pre-trained VGG16 network for transfer learning. (a) VGG16's training accuracy; (b) VGG16's training loss.

**Figure 7:**
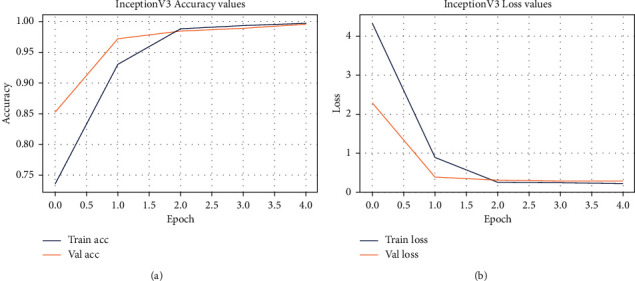
Pre-trained InceptionV3 network for transfer learning. (a) InceptionV3's training accuracy; (b) InceptionV3's training loss.

The accuracy/loss subplot shows the continuous learning of a model. Selected models were tested for 5 epochs in this experiment.  Figure 6(a) and Figure 7(a) show training and validation accuracy graph, whereas Figure 6(b) and Figure 7(b) show training and validation loss graph of implemented models. In graph, blue and red lines show training accuracy/loss and validation accuracy/loss of the selected models, respectively.

*VGG16 Training Accuracy/Loss*. According to Figure 6, VGG16 has achieved a maximum accuracy of 98% with a training loss of 0.08, that is, model learned effectively and properly distinguish between spondylolisthesis and normal cases.

*InceptionV3 Training Accuracy/Loss*.  Figure 7 indicates that InceptionV3 has achieved 96% accuracy with a training loss of 0.08. It shows that our model has learned effectively but it is less accurate to classify between spondylolisthesis and normal cases.

#### 5.2.2. Confusion Matrix

**Figure 8:**
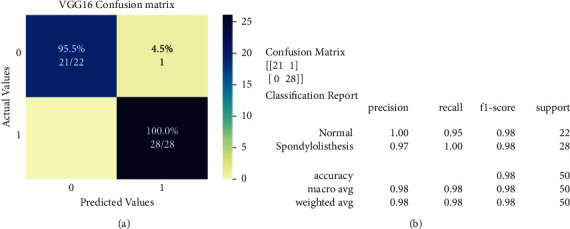
VGG16's confusion matrix and classification report. (a) VGG16's confusion matrix; (b) VGG16's classification report.

**Figure 9:**
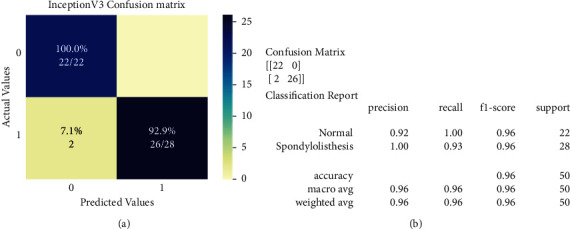
InceptionV3's confusion matrix and classification report. (a) InceptionV3's confusion matrix; (b) InceptionV3's classification report.

Confusion matrices of VGG16 and InceptionV3 models are displayed in Figure 8(a) and Figure 9(a), respectively. A total of 50 X-ray radiographs were used in the test set (28 spondylolisthesis and 22 normal). In the confusion matrix, actual cases were arranged in rows, whereas predicted cases were arranged in columns. Also, class 0 and class 1 indicate normal and spondylolisthesis cases, respectively.

*VGG16's Confusion Matrix and Classification Report*. In the context of VGG16 (Figure 8(a)), out of 22 normal patients, the model correctly identified 21 and misclassified 1 case as spondylolisthesis. All typical instances had their precise class label predicted by the model.

*InceptionV3's Confusion Matrix and Classification Report*. In the case of InceptionV3 (Figure 9(a)), the model correctly identified 26 of 28 spondylolisthesis patients and misclassified 2 cases as normal. The model correctly identified all the normal cases.

#### 5.2.3. Assessment of Performance Using Metrics

Accuracy, precision, recall, and F1-score were utilized to evaluate the performance of selected models in this work. These metrics are calculated using the following formulae.

Accuracy: the number of correct predictions divided by the total number of predictions is known as accuracy.

(1) $$\begin{array}{c} \text{Accuracy}=\frac{\left(TP+TN\right)}{\left(TP+FP+TN+FN\right)}. \end{array}$$

Precision: the accuracy of the prediction is measured by precision.

(2) $$\begin{array}{c} \text{Pricision}=\;\frac{TP}{\left(TP+FP\right)}. \end{array}$$

Recall: the recall of the detector is measured by how well it discovers all ground truth.

(3) $$\begin{array}{c} \text{Recall}=\;\frac{TP}{\left(TP+FN\right)}. \end{array}$$

F1 score: when you need a quick way to compare two classifiers, it is frequently easier to combine accuracy and recall into a single statistic called the F1-score. The harmonic mean of precision and recall is used to get the F1-score.

(4) $$\begin{array}{c} F1\text{ Score}=2*\frac{\left(\text{Precision}*\text{Recall}\right)}{\left(\text{Precision}+\text{Recall}\right)}. \end{array}$$

Table 5 shows the precision, recall, accuracy, and F1-score (described in equation [1,4]) and Figure 8(b) and Figure 9(b) show the graphical representation of selected classifiers.

#### 5.2.4. Model Compression Using TFLite

VGG16 and InceptionV3 models were compressed up to four times for use on small devices. A helper function is used to evaluate the performance of the compressed model on the test dataset.  Table 6 illustrates the comparison between original model and compressed model.

### 5.3. Performance Comparison Using Publicly Available Dataset

**Figure 10:**
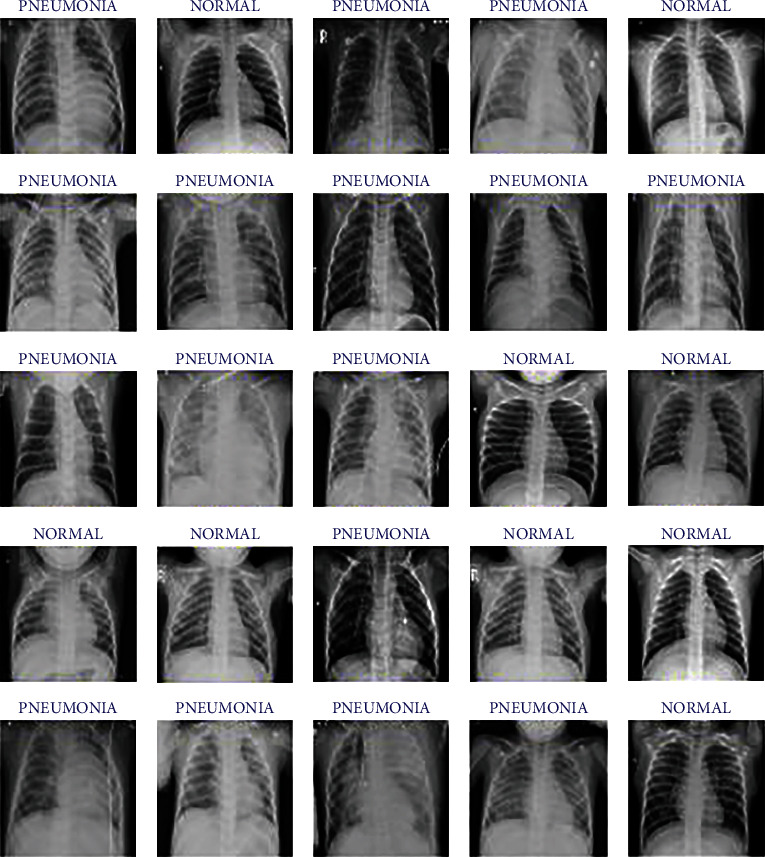
Some images from Kaggle's Pneumonia dataset.

Kaggle's Pneumonia dataset is used to compare the findings of our outperformed model. Some samples from the selected dataset are displayed in Figure 10.

Out of 5232 images, 3883 images are of Pneumonia patients and 1349 images are of normal patients. Using a conventional 70 : 30 ratio, the dataset is separated into training and test sets, and the test set is further segmented into test and validation sets. The statistics of the split dataset are described in Table 7.

#### 5.3.1. Training Accuracy/Loss

**Figure 11:**
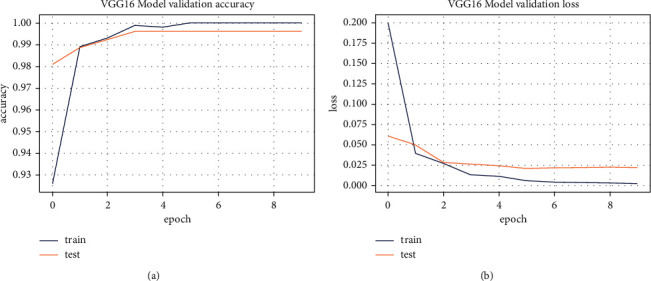
Training accuracy/loss graph of Pneumonia dataset using VGG16. (a) Training accuracy. (b) Training loss.

The training accuracy/loss graph for the outperformed model (VGG16) is shown in Figure 11.

#### 5.3.2. Confusion Matrix

**Figure 12:**
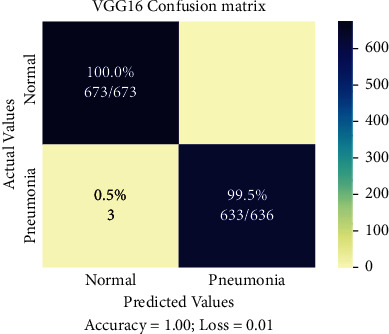
Confusion matrix of Pneumonia dataset.

Confusion matrix of a selected Pneumonia dataset is shown in Figure 12. It is self-evident that the VGG16 model correctly categorised the cases as normal and Pneumonia patients.

#### 5.3.3. Compressing the Model Using TFLite

The fine-tuned VGG16 model is compressed four times to form a quantized model.  Table 8 shows the size and accuracy of the base and quantized models.

According to above table, the quantized model has achieved 100% accuracy which validates our previous finding. The implemented quantized model worked similarly for both the private (spondylolisthesis) and public (Pneumonia) datasets.

## 6. Result Analysis and Discussion

In this study, two pre-trained transfer learning CNN models, VGG16 and InceptionV3, were selected for the disease classification task. Spine X-ray radiographs of normal and spondylolisthesis patients were used to train, validate, and test these models. The data augmentation technique is used to create enough images (total of 1000 samples). The goal was to accurately diagnose the disease and assess the performance of the selected model. According to above figures and tables, VGG16 and InceptionV3 have achieved 98% and 96% accuracy rates, respectively.

In prior study, Varcin et al. [23] have employed two distinct networks, AlexNet and GoogLeNet, for spondylolisthesis diagnosis on their private datasets. According to the research, GoogLeNet is somewhat more successful than AlexNet by attaining 93% accuracy rate. Our results outperform the prior work by attaining a peak accuracy of 98%.

Both models were compressed up to four times using TFLite converter. Our finding shows that there is minor (2% increase in case of VGG16) or no difference (in case of InceptionV3) in accuracies of original model and quantized model. According to literature survey (Table 1), the suggested model VGG16 + TFLite attained 100% accuracy, which is higher than Zebin and Rezvy's [29] 90% accuracy for the same model.

The model has been applied to a publicly available dataset. Pneumonia dataset has a larger amount of data than our spondylolisthesis dataset, and the accuracy attained by the Pneumonia's VGG16 model is higher than spondylolisthesis's VGG16 model. On the basis of our findings, we can conclude that implemented quantized model is more reliable and efficient for disease classification in general.

## 7. Conclusion

In this study, the performances of two deep neural networks, VGG16 and InceptionV3, were compared for spondylolisthesis diagnosis. Data augmentation is used to increase the sample size. VGG16 model has achieved 98% accuracy rate, which is higher than InceptionV3's 96% accuracy rate. Also, we have applied quantization to reduce the model size up to four times. The implemented models outperformed prior studies. Moreover, we have generalized the model on the public platform.

Although these models may be used as a substitute for manual radiological analysis and can help clinicians to diagnose spondylolisthesis from spine X-ray data automatically, further study is needed for grading spondylolisthesis through X-ray images.

## Figures and Tables

**Table 1:** Summary of literature review.

Source	Purpose	Major findings	Accuracy (%)
Varcin et al. [23]	Diagnosis of lumbar spondylolisthesis	AlexNet and GoogLeNet were used for spondylolisthesis diagnosis. The model is not suitable in terms of accuracy.	AlexNet: 93.87
			GoogLeNet: 91.67
CoCoci et al. [24]	Pneumonia detection on chest X-ray	MobileNetV3, ShuffleNetV2, and SlimNet models with Android implementations in TensorFlow Lite are presented for constructing intelligent medical devices.	MobileNetV3: 95.9
			ShuffleNetV2: 96.67
			SlimNet: 96.83
Cococi et al. [25]	Disease detection from chest X-ray	A sophisticated medical device is built with an Android and Raspberry Pi-based strategy.	91.22
Basantwani et al. [26]	COVID-19 detection from chest X-rays and CT scans	Android app was built to convert the final model into a TFLite model which could be used in making the Android model	94
Verma et al. [27]	Detecting COVID-19 from chest CT scans	Model's size is reduced by utilising TensorFlow lite, and model is tuned for speed and latency on edge devices.	99.58
Bushra et al. [28]	Detection of COVID-19 from X-ray images	An Android application is developed which uses the TFLite model	98.65
Zebin and Rezvy [29]	Detection of COVID-19 using chest X-ray	Multiple pretrained models were used for detection of chest disease from X-ray images.	VGG16: 90
			ResNet50: 94.3
			EfficientNetB0: 96.8
Sharma et al. [30]	Multilabel classification of retinal disorders using OCT	Deep-learning-based detection method for screening people with blinding retinal diseases is proposed which can be remedied if detected early.	99.38

**Table 2:** Some features of selected pre-trained model.

Network	Year	Depth	Architecture	Parameters (M)
VGG16	2014	23	Classic network	138
InceptionV3	2015	159	Modern network	24

**Table 3:** Dataset description.

Test cases	299
Normal	143
Spondylolisthesis	156
Image dimension	224 × 224 × 3
Image type	X-ray radiograph (.jpg format)

**Table 4:** Dataset statistics.

Test cases	Training set	Test set	Validation set
Normal	210	22	75
Spondylolisthesis	490	28	175

Total	700	50	250

**Table 5:** Learning outcomes.

Model/performance metrics	Accuracy	Precision	Recall	F1-score	Loss
VGG16	0.98	0.97	1.00	0.98	0.08
InceptionV3	0.96	1.00	0.93	0.96	0.08

**Table 6:** 4x compression of implemented model.

Model name	TFLite model size		TFLite model accuracy	
	Base model	Quantized model	Base model	Quantized model
VGG16	59068092	14871680	0.98	0.1
InceptionV3	87533216	22325120	0.96	0.96

**Table 7:** Pneumonia dataset statistics.

Test cases	Training set	Test set	Validation set
Normal	944	338	67
Pneumonia	2718	971	194

Total	3662	1309	261

**Table 8:** VGG16 model 4x compression.

Model name	TFLite model size		TFLite model accuracy	
	Base model	Quantized model	Base model	Quantized model
VGG16	59067384	14870960	0.997	0.1
